# Shiga toxin signals via ATP and its effect is blocked by purinergic receptor antagonism

**DOI:** 10.1038/s41598-019-50692-1

**Published:** 2019-10-07

**Authors:** Karl E. Johansson, Anne-Lie Ståhl, Ida Arvidsson, Sebastian Loos, Ashmita Tontanahal, Johan Rebetz, Milan Chromek, Ann-Charlotte Kristoffersson, Ludger Johannes, Diana Karpman

**Affiliations:** 1 Department of Pediatrics, Clinical Sciences Lund, Lund University, Lund, Sweden; 2 Institut Curie, U1143 INSERM, UMR3666 CNRS, Cellular and Chemical Biology unit, Paris, France

**Keywords:** Bacteriology, Haemolytic uraemic syndrome, Bacteriology, Haemolytic uraemic syndrome

## Abstract

Shiga toxin (Stx) is the main virulence factor of enterohemorrhagic *Escherichia coli* (EHEC), that cause gastrointestinal infection leading to hemolytic uremic syndrome. The aim of this study was to investigate if Stx signals via ATP and if blockade of purinergic receptors could be protective. Stx induced ATP release from HeLa cells and in a mouse model. Toxin induced rapid calcium influx into HeLa cells, as well as platelets, and a P2X1 receptor antagonist, NF449, abolished this effect. Likewise, the P2X antagonist suramin blocked calcium influx in Hela cells. NF449 did not affect toxin intracellular retrograde transport, however, cells pre-treated with NF449 exhibited significantly higher viability after exposure to Stx for 24 hours, compared to untreated cells. NF449 protected HeLa cells from protein synthesis inhibition and from Stx-induced apoptosis, assayed by caspase 3/7 activity. The latter effect was confirmed by P2X1 receptor silencing. Stx induced the release of toxin-positive HeLa cell- and platelet-derived microvesicles, detected by flow cytometry, an effect significantly reduced by NF449 or suramin. Suramin decreased microvesicle levels in mice injected with Stx or inoculated with Stx-producing EHEC. Taken together, we describe a novel mechanism of Stx-mediated cellular injury associated with ATP signaling and inhibited by P2X receptor blockade.

## Introduction

Shiga toxin (Stx) is the main virulence factor of enterohemorrhagic *Escherichia coli* (EHEC). These strains are causally associated with hemolytic uremic syndrome (HUS), a major cause of acute renal failure. There are two major variants of Stxs, Stx1 and Stx2, that are approximately 60% homologous^1^. The toxin consists of one enzymatically active A-subunit and a pentameric B-subunit^2,3^. The Stx B-subunit binds to the glycolipid receptor globotriaosylceramide (Gb3) or globotetraosylceramide (Gb4)^4^, leading to internalization of the toxin^5^. Once endocytosed, Stx undergoes retrograde transport via the Golgi apparatus to the endoplasmic reticulum. During retrograde transport the A-subunit is cleaved by furin into A_1_ and A_2_ fragments^6^. From the ER the A_1_ fragment is released into the cytosol where it depurinates an adenine base from the 28S rRNA of the ribosome^3^, thereby inhibiting protein synthesis and subsequently leading to cell death^7,8^. Stx induces apoptosis in intestinal^9^ and kidney^10^ cells *in vivo* and also in HeLa cells *in vitro*^11^.

Upon receptor binding, Stx1 and Stx2 can trigger cellular activation and host responses (reviewed in^12,13^). Stx1 has been shown to induce calcium influx into HeLa cells^14^. Furthermore, Stx2 stimulates microvesicle shedding from activated blood cells^15–18^. Microvesicles are extracellular vesicles, shed directly from the plasma membrane^19^. Their formation is a regulated process initiated by elevation in intracellular calcium levels leading to loss of plasma membrane lipid asymmetry and cleavage of cortical actin^20^. Microvesicles act as carriers of proteins, lipids and nucleic acids, delivering their cargo to target cells^21^. Importantly, in the context of Stx-mediated HUS, microvesicles transfer the toxin to the kidney^16^.

Purinergic receptors are a family of trans-membrane receptors sub-divided into P1 and P2 receptors, based on ligand binding. P1 receptors are activated by adenosine and P2 receptors are activated by ATP and other purine nucleotides^22^. P2 receptors are further subdivided into ionotropic P2X and metabotropic P2Y receptors^23^ and the P2X1 receptor is a known calcium channel in platelets^24^.

Here we investigated if Stx-induces cellular activation and damage via ATP signaling and, if so, if injury could be decreased by blockade of purinergic signaling. To this end, we used HeLa cells and platelets incubated with the P2X1 specific antagonist NF449^25^ and the non-selective P2X antagonist suramin^26^ and P2X1-deficient HeLa cells, to determine the effect of purinergic signaling on Stx-mediated calcium influx, toxin retrograde transport, cytotoxicity and microvesicle release. We used an established mouse model^27,28^, in which mice were injected with Stx, to study ATP release, and investigated the effect of purinergic receptor blockade on microvesicle shedding in mice injected with Stx2 or inoculated with Stx2-producing *E. coli*.

## Results

### Stx induced release of ATP *in vitro* and *in vivo*

**Figure 1:**
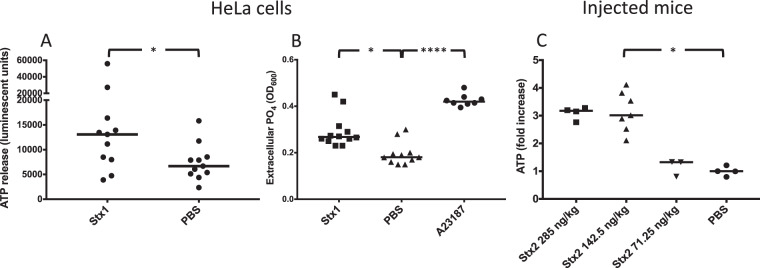
Shiga toxin induces release of ATP *in vitro* and *in vivo*. (**A**) HeLa cells (n = 11) were stimulated with Shiga toxin 1 (Stx1) or PBS and the ATP content was measured after 5 min. The median extracellular ATP content is depicted as the bars. (**B**) HeLa cells were stimulated with Stx1 (n = 12), A23187 calcium ionophore (n = 8) or PBS (n = 11). The supernatant was collected and free phosphate groups were measured. Data is depicted as median absorbance at OD_600_ (denoted by the bars), correlating to free phosphate groups in the supernatant. (**C**) Mice were injected with Stx2 at a concentration of 285 (n = 4), 142.5 (n = 7) or 71.25 ng/kg (n = 3) or with PBS as the control. Mice injected with Stx2 142.5 ng/kg had significantly higher plasma ATP levels compared to PBS control mice. A similar trend was seen for mice injected with Stx2 285 ng/kg, although the difference did not achieve statistical significance. Mice injected with Stx2 71.25 ng/kg had plasma ATP levels comparable to PBS control mice. The median is denoted by the bar. *P < 0.05, ****P < 0.0001, two-tailed Mann-Whitney test (panel A) and Kruskal-Wallis test (panels B and C).

HeLa cells were stimulated with Stx1 (1 μg/mL). These cells were chosen because they possess the toxin receptor Gb3 and are susceptible to the toxin^14,29^. ATP was released from HeLa cells stimulated with Stx1 for 5 min. A significant increase in extracellular ATP was detected in the medium of toxin-stimulated cells compared to the phosphate buffered saline (PBS) control (Fig. 1A). The rapid ATP release induced by Stx1 indicated that toxin binding in itself led to the response. A lower concentration of Stx1 (200 ng/mL) also induced the release of extracellular ATP that reached significance after 10 min incubation (Supplementary Fig. S1A). A similar trend, albeit non-significant, was noted when cells were stimulated with Stx2 (1 μg/mL) (Supplementary Fig. S1B). The positive control, histamine, led to rapid ATP release (median 33240 luminescent units).

Free extracellular phosphate groups served as an indicator of ATP degradation after release. A significant increase in phosphate groups was observed in the medium from Stx1-stimulated cells and calcium-ionophore stimulated cells (the positive control) compared to the PBS negative control after 40 min (Fig. 1B), indicating ATP degradation. The data suggest that Stx1 released ATP from HeLa cells and that the signal was degraded extracellularly.

ATP content was analyzed in plasma samples from mice that had been injected with varying doses of Stx2 and sacrificed when symptoms developed. Stx2 was chosen for *in vivo* experiments as its toxicity in murine disease has been previously demonstrated^27^. Mice treated with Stx2 at a dose of 285 ng/kg developed symptoms on day 3 after injection, those treated with Stx2 142.5 ng/kg developed symptoms on day 4 or 5 and mice treated with the lowest dose (71.25 ng/kg) remained asymptomatic. Plasma ATP was significantly higher in symptomatic toxin-injected mice (Stx2 142.5 ng/kg, Fig. 1C). Mice treated with the lowest dose of Stx2 had ATP levels comparable to untreated mice.

### P2X1 receptor antagonist inhibited Stx1 and Stx2-induced calcium influx

**Figure 2:**
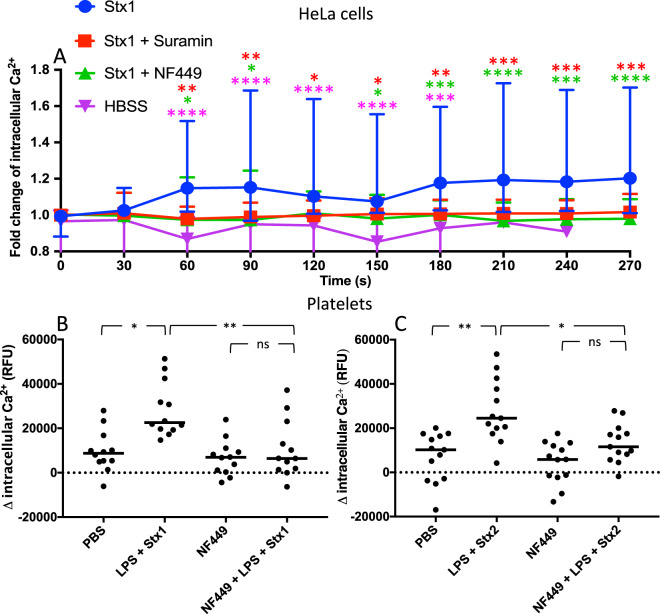
The effect of purinergic antagonists on calcium influx induced by Shiga toxin in HeLa cells and human platelets. (**A**) Calcium influx was measured in HeLa cells preincubated with NF449, suramin or phosphate buffered saline (PBS) vehicle, stimulated with Shiga toxin 1 (Stx1) or Hank’s balanced salt solution (HBSS) (groups differentiated by icon colors) and imaged by fluorescence microscopy. Results are presented as mean fluorescent change of all cells in the field of view (median and range). The color of the asterisks corresponds to the color of the icon in comparison to Stx1. The absence of asterisks indicates that statistics was not significant. (**B-C**) Human platelets (n = 3 donors) were preincubated with NF449 or PBS vehicle followed by Stx1 (**B**) or Stx2 (**C**) and O157LPS (to enable platelet activation by Shiga toxin) or PBS vehicle. Data is presented as the initial fluorescence subtracted from fluorescence after 2 minutes and the bar denotes the median fluorescence. RFU: relative fluorescent units, ns: not significant, *P < 0.05, **P < 0.01, ***P < 0.001, ****P < 0.0001, two-way repeated measure ANOVA (panel A) and Kruskal-Wallis test (panels B and C).

To evaluate the importance of Stx-induced ATP-release for Stx1-mediated signaling, experiments were carried out to study if the P2X1 antagonist NF449, or the non-selective P2X inhibitor suramin, could block calcium influx induced by Stx1. HeLa cells loaded with Fluo-4 calcium indicator dye and stimulated with Stx1 displayed a swift and steady increase in cytosolic calcium, lasting for the duration of the experiment, 270 sec (Fig. 2A). NF449- and suramin-pretreated cells exhibited significantly less calcium influx after Stx1 stimulation compared to untreated cells, remaining at stable low calcium concentration levels throughout the experiment (Fig. 2A) as did the HBSS negative control. As a positive control, NF449 treated and untreated HeLa cells were stimulated with ATP. ATP induced a clear calcium response in HeLa cells, while NF449 treated cells were unaffected (Supplementary Fig. S2).

A similar experiment was carried out using human platelets stimulated with Stx1 or Stx2, together with *E. coli* O157 lipopolysaccharide (LPS) to stimulate platelet activation^18,30^. An increase in intracellular calcium levels was noted upon Stx1 (Fig. 2B) and Stx2 (Fig. 2C) stimulation. When the cells were pre-treated with NF449 calcium influx was not detected (Fig. 2B,2C). Stx1, Stx2 or O157LPS alone did not have a significant effect on the influx of calcium (Supplementary Fig. S3A,B).

### Stx1 localization to the ER was not affected by the P2X1 receptor antagonist

**Figure 3:**
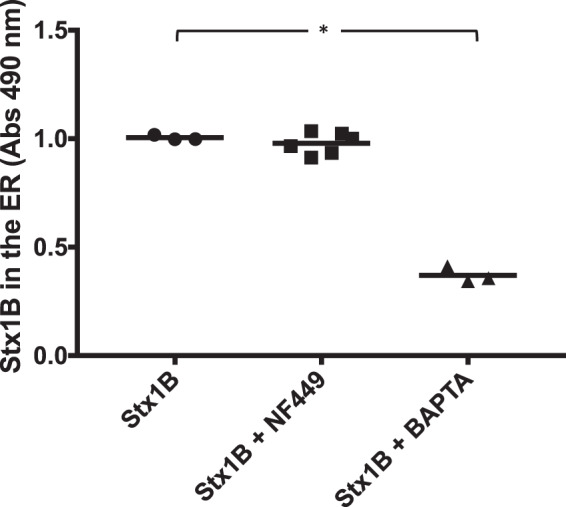
The effect of NF449 on retrograde transport of Shiga toxin 1B. HeLa cells transfected with the ER-anchored SNAP-tag were treated with NF449 (n = 6), the Ca^2+^ chelator BAPTA-AM (n = 3) or PBS vehicle (n = 3) followed by Shiga toxin (Stx) 1B-subunit:O^6^-benzylguanine. No difference in Stx1B localization to the ER could be seen between NF449-treated and PBS-treated cells. Data is shown as median absorbance values (OD_490_) from SNAP-captured Stx1B. The median is depicted by the bar. *P < 0.05, n.s.: non-significant, Kruskal-Wallis test.

The effect of the P2X1 receptor antagonist NF449 on Stx1-retrograde transport was studied using HeLa cells expressing a SNAP-tag localized to the ER that were incubated with Stx1B labeled with the binding substance O^6^-benzylguanine. NF449 did not affect retrograde trafficking of the toxin to the ER whereas the positive control, the Ca^2+^ chelator BAPTA-AM, resulted in significantly less toxin locating to the ER (Fig. 3).

### P2X1 receptor antagonist protected HeLa cells from the cytotoxic effects of Stx

**Figure 4:**
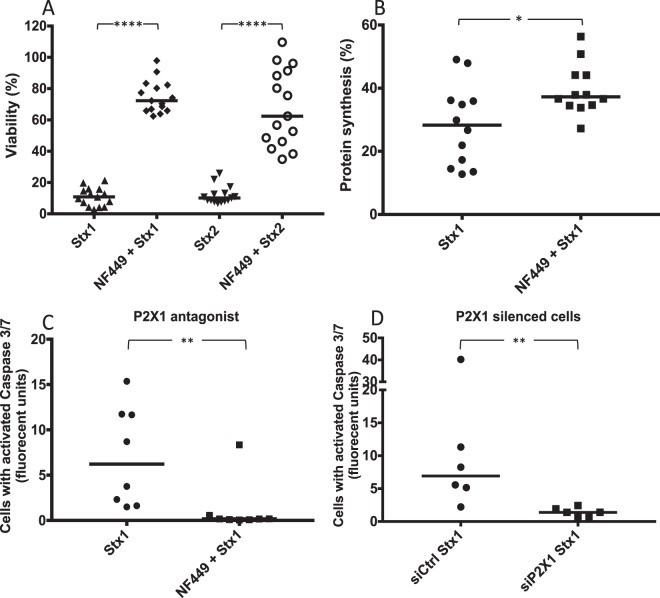
The effect of NF449 on HeLa cell viability upon Shiga toxin intoxication. (**A**) PBS-treated and NF449-treated HeLa cells were incubated with Shiga toxin (Stx)1 or Stx2 for 24h. NF449-treated cells exhibited 61.5% and 52.3% less cell death compared to PBS-treated cells (defined as 100%) when challenged with Stx1 and Stx2, respectively. Median viability is depicted by the bar. (**B**) HeLa cells treated with NF449 or PBS vehicle were incubated with Stx1 and protein synthesis was measured. PBS-treated cells displayed a lower protein synthesis (median 28%) compared to cells pretreated with NF449 (median 37%). Protein synthesis is presented as percent ^35^S divided by total protein content compared to the toxin-free control (defined as 100%). The median protein synthesis is depicted as the bar. (**C**) Stx1-induced caspase 3/7 activation was measured in HeLa cells pretreated with NF449 or left untreated, showing less caspase 3/7 activation in the cells that were pretreated with NF449. (**D**) HeLa cells were transfected with siP2X1 or siCtrl (n = 6) and challenged with Stx1, showing less caspase 3/7 activation in cells transfected with siP2X1. Median caspase 3/7 activation per cell is denoted by the bar. *P < 0.05, **P < 0.01, ****P < 0.0001, two-tailed Mann-Whitney test.

Stx1- and Stx2-treated HeLa cells exhibited considerable cell death, a median of 10.8% (range 2.7–21.3%) and 10.1% (range 6.8–25.9%), respectively, were viable after 24 h. Cell viability was significantly higher in cells that were pre-treated with NF449 with a median of 72.3% (range 63.9–97.9%) for Stx1 and 62.4% (34.9–109.6%) for Stx2 compared to PBS-treated cells, defined as 100% viability (Fig. 4A).

### P2X1 receptor antagonist partially protected HeLa cells from Stx1-mediated ribotoxicity

The effect of Stx1 on protein synthesis was investigated in the presence of NF449. HeLa cells incubated with Stx1 alone for 4 h displayed a median of 28% (range 13–49%) protein synthesis compared to unstimulated control cells (defined as 100%). Pre-incubation with NF449 increased protein synthesis to 37% (range 27–56%) compared to unstimulated cells (Fig. 4B).

### P2X1 receptor antagonist reduced Stx1-induced caspase 3/7 activation

Stx1 was incubated with HeLa cells for 24 h and induced caspase 3/7 activation in a majority of the cells, suggesting the induction of apoptotic signals (Fig. 4C). Pretreatment with NF449 lead to significantly less caspase 3/7 activation per cell (Fig. 4C). Similar results were obtained with Stx2 (Supplementary Fig. S4).

Cells with reduced P2X1 receptor expression, due to silencing, displayed significantly lower caspase-3/7 activation in response to Stx1 compared to control cells transfected with a scrambled sequence (Fig. 4D), supporting involvement of the receptor. PBS-treated native (n = 3) and P2X1-silenced HeLa cells (n = 2) did not display caspase 3/7-activation (data not shown).

### P2X receptor antagonists decreased the release of Stx1 and Stx2-positive microvesicles

**Figure 5:**
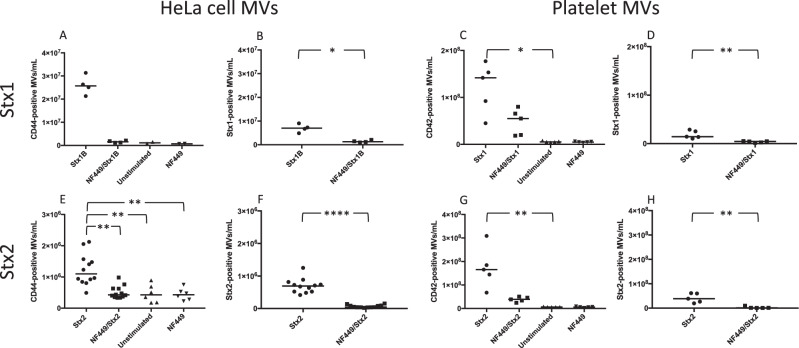
NF449 inhibits the release of toxin-positive microvesicles from HeLa cells and platelets. (**A–B**) HeLa cells were pretreated NF449 or left untreated and stimulated with Shiga toxin 1B (Stx1B). Microvesicle release was measured by flow cytometry. (**A**) Stx1B induced the release of microvesicles from HeLa cells (CD44 + ), the effect was not significant compared to NF449 pretreated cells (median microvesicles (MVs) in NF449 control was 7.3 × 10^5^/mL). (**B**) The release of toxin-positive microvesicles (those containing Stx1B) was significantly decreased by NF449 (median MVs in NF449/Stx1B was 1.3 × 10^6^/mL). (**C–D**) Whole blood was pretreated with NF449 or left untreated and stimulated with Stx1 (holotoxin). (**C**) Stx1 induced a significant release of platelet-derived microvesicles (CD42 + , median MVs in NF449 control was 4.8 × 10^6^/mL). (**D**) The release of toxin-positive platelet microvesicles was significantly reduced in the presence of NF449 (median MVs in NF449/Stx1 was 4.4 × 10^6^/mL). (**E–F**) HeLa cells were pretreated with NF449 or left untreated and stimulated with Stx2 (holotoxin). (**E**) Stx2 induced a significant release of microvesicles that was significantly reduced by NF449 (median MVs in NF449 control was 3.8 × 10^5^/mL). (**F**) Toxin-positive microvesicle release was significantly reduced by NF449 (median MVs in NF449/Stx2 was 4.7 × 10^4^/mL). (**G–H**) Whole blood was pretreated with NF449 or left untreated and stimulated with Stx2. (**G**) Stx2 induced a significant release of platelet-derived microvesicles (median MVs in unstimulated control was 6.3 × 10^6^/mL). (**H**) NF449 significantly reduced the release of Stx2-positive microvesicles from platelets (median MVs in NF449/stx2 was 1 × 10^6^/mL). Median microvesicles/mL is denoted as the bar. *P < 0.05, **P < 0.01, ****P < 0.0001, two-tailed Mann-Whitney test (panels B, D, F and H) and Kruskal-Wallis test (panels A, C, E and G).

Calcium influx induces microvesicle release^31^ and, as shown above, Stx triggers calcium influx, which has also been demonstrated for Stx1B^14^. Stx1B stimulation induced microvesicle release from HeLa cells that was reduced in the presence of NF449, although this did not achieve statistical significance (Fig. 5A). HeLa cell-derived microvesicles containing Stx1B were significantly reduced in the presence of NF449 (Fig. 5B). Experiments were also carried out in whole blood in which Stx1 (holotoxin) induced a significant release of microvesicles from platelets (Fig. 5C) and NF449 significantly reduced the release of Stx1-positive platelet microvesicles (Fig. 5D). Similar experiments were carried out with Stx2 (holotoxin). Stx2 stimulation induced the release of HeLa cell microvesicles, an effect that was significantly reduced by NF449 (Fig. 5E). Likewise, the release of toxin-positive microvesicles was significantly decreased by NF449 (Fig. 5F). Platelet-derived microvesicles were released in whole blood stimulated with Stx2 (Fig. 5G), this effect was significantly reduced by suramin (Supplementary Fig. S5) and by NF449, albeit without achieving significance (Fig. 5G). Significantly less Stx2-positive platelet microvesicles were released in the presence of NF449 (Fig. 5H).

### Suramin inhibits Stx2-induced platelet-derived microvesicle release *in vivo* in a Stx2 and EHEC mouse model

**Figure 6:**
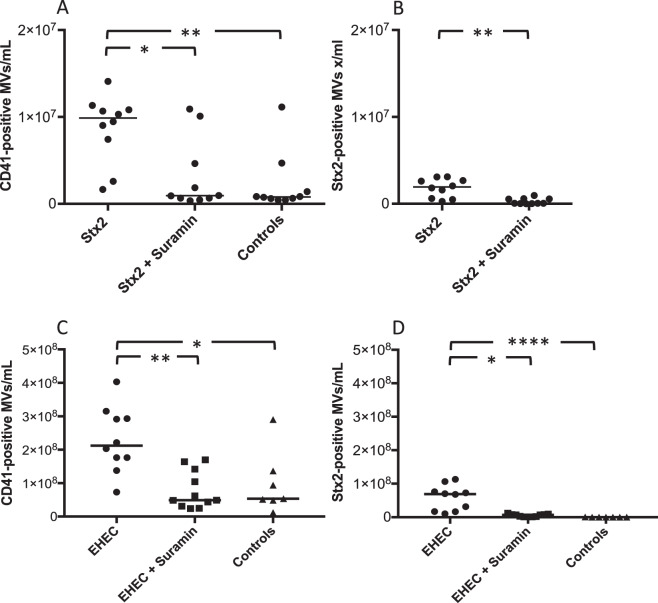
Suramin inhibits Shiga toxin 2- and EHEC-induced platelet-derived microvesicles and Shiga toxin 2-positive microvesicle-release *in vivo*. (**A** and **B**) Mice were pretreated with suramin or vehicle followed by injection of Shiga toxin 2 (Stx2) or PBS control (n = 10). (**A**) Stx2-injected mice had significantly higher levels of circulating platelet-derived microvesicles (MV, CD41+) and suramin pretreatment reduced this effect. (**B**) Stx2-positive microvesicles were significantly lower in mice pretreated with suramin. (**C** and **D**) Mice were inoculated with EHEC (n = 10) and certain mice were pretreated with suramin (n = 11) or inoculated with vehicle (n = 7). (**C**) EHEC-inoculated mice had significantly higher levels of circulating platelet-derived microvesicles compared to mice pretreated with suramin and PBS-treated mice. (**D**) Stx2-positive microvesicles were significantly higher in EHEC-inoculated mice and suramin pretreatment significantly lowered this effect. Median microvesicles/mL is denoted by the bar. *P < 0.05, **P < 0.01, ****P < 0.0001, two-tailed Mann-Whitney test (panel B) and Kruskal-Wallis test (panel A, C and D).

The effect of suramin treatment on platelet-derived microvesicles, and the release of Stx2-positive microvesicles, was assessed in BALB/c mice injected with Stx2. Suramin was chosen for the *in vivo* studies due to its non-selective antagonistic properties with regard to P2X receptors^32^. Levels of platelet-derived microvesicles were significantly higher in mice injected with Stx2 compared to controls (Fig. 6A). Platelet-derived microvesicles and those that were Stx2-positive were significantly lower in suramin-treated mice (Fig. 6A,B).

The effect of suramin treatment was assessed in BALB/c mice infected with Stx2-producing *E. coli* O157:H7. The number of total platelet-derived microvesicles, and those that were Stx2-positive, in plasma from mice infected with *E. coli* O157:H7 was significantly higher compared to the control mice (inoculated with vehicle) and suramin treatment significantly reduced this effect (Fig. 6C,6D).

## Discussion

**Figure 7:**
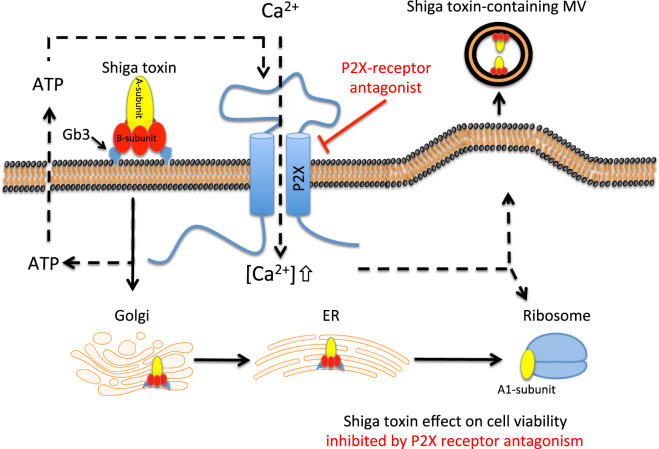
Proposed mechanism of Shiga toxin induced cellular activation via ATP. Schematic diagram displaying a proposed mechanism by which Shiga toxin induces cellular activation, utilizing ATP as a second messenger. When Shiga toxin binds to its receptor, globotriaosylceramide (Gb3), cells release ATP. ATP in turn binds to P2X receptors causing Ca^2+^-influx into the cell. Ca^2+^ is necessary for intracellular processes associated with Shiga toxin induced cell death and the shedding of toxin-positive microvesicles (MVs). Purinergic receptor blockade, such as NF449 and suramin, used in this study, block P2X-activation and these Shiga toxin-mediated effects. Filled arrows show the transport route of Shiga toxin and dashed arrows show ATP-mediated signaling. ER: endoplasmic reticulum. This illustration was created using software from Motifolio.

Stx, the major virulence factor of EHEC strains, causes massive intestinal and renal cellular damage. This study demonstrates a novel mechanism by which Stx activates and damages cells. Stx induces a cellular signal promoting the release of ATP followed by ATP signaling via purinergic receptors, leading to calcium influx into cells associated with toxin-associated cellular damage and microvesicle release. Stx mediates cellular injury by binding to the Gb3 receptor and undergoing cellular uptake, retrograde transport and thereafter causing cell death by inhibition of protein synthesis and induction of apoptosis. The toxin gains access to the circulation, binds to blood cells and induces the release of blood cell-derived microvesicles^33^. Stx reaches the kidney bound to the cell membrane of blood cells or within blood cell-derived microvesicles^16^. This study shows that ATP signaling is involved in fundamental aspects of Stx-mediated cellular effects as depicted in Fig. 7. This novel ATP-mediated effect of Stx was inhibited by the P2X1 antagonist NF449, and the non-selective antagonist suramin. NF449 blocked toxin-associated calcium influx and reduced cytotoxicity, associated with inhibited protein synthesis and caspase activity, as well as microvesicle release. The latter effect, on microvesicle release, was confirmed in two *in vivo* models using Stx2 injection and *E. coli* O157 inoculation. Purinergic antagonism could thus block crucial aspects of Stx-induced cellular activation and injury. The findings suggest that ATP signaling can promote the cellular effects of Stx and that blocking purinergic receptors may have a protective effect.

Stx1 induces calcium influx^14^. The mechanism by which the toxin induces calcium influx has not been previously elucidated. Here we show that toxin induces ATP efflux, which then binds to and activates the P2X1 receptor triggering calcium influx that could be demonstrated for both Stx1 and Stx2. The effect of Stx2 was specifically assayed in the presence of apyrase, in order to catalyze the hydrolysis of preformed ATP to ADP and AMP and thereby reduce P2X1 desensitization^34^. This effect of Stx was totally abrogated by the P2X1 receptor antagonist NF449 in HeLa cells (for Stx1) and in platelets (for Stx1 and Stx2). We used HeLa cells throughout the study to obtain reproducible results not dependent on variations between donors and showed that the cells express the P2X1 receptor. We chose to show the effect of Stx1 and Stx2 on ATP signaling even in platelets as these cells are known to express P2X1^35^ and signaling via the P2X1 receptor was shown to induce calcium influx in platelets (reviewed in^24^). Moreover, once Stx gains access to the bloodstream it binds to platelets^18^, thus the signaling induced in platelets, and its abrogation by NF449, may have relevance in the clinical disease setting.

Blocked ATP signaling and calcium influx affected the ultimate cellular effects of Stx1, i.e. the induction of cell death by inhibited protein translation and apoptosis. These effects were reduced in the presence of the P2X1 receptor antagonist NF449. Calcium flux has been implicated in the induction of apoptosis (reviewed in^36^). Ribosome-inactivating proteins, such as ricin and Stx, of plant or microbial origin, respectively, require intracellular calcium to induce apoptosis^37^. Calcium transfer from the ER to mitochondria induced by Stx1, leading to release of cytochrome *c*, activation of caspase-9 and production of reactive oxygen species, caused cell death by apoptosis^11,38^. Moreover, calpain activity, involved in Stx1-mediated caspase-8 cleavage and apoptosis, is calcium-dependent^39^. The ribosome-inactivating RNA *N*-glycosidase activity of ricin was shown to be divalent cation-dependent^40^. Taken together, the effects of Stx1 on inhibition of protein synthesis and the induction of apoptosis are calcium-dependent, thus NF449 could affect these intracellular pathways by inhibiting calcium influx, thereby exerting a protective effect.

In calcium-free medium Stx1B does not induce calcium influx^14^, indicating that the elevated calcium originates from an extracellular source. NF449 did not affect the transport of the toxin to the ER, while BAPTA-AM (the positive control) did (Fig. 3). BAPTA-AM is a cell permeable version of BAPTA that can chelate intracellular divalent cations with high specificity for Ca^2+^, leaving minimal levels of free intracellular Ca^2+^, whereas NF449 affects Ca^2+^ influx into cells, but not intracellular Ca^2+^ sources. As NF449 does not perturb intracellular Ca^2+^ sources this may explain why toxin transport to the ER was not affected.

The P2X receptors differ in the number of their subunits, from 1 to 7, and these subunits share a certain degree of homology^41^. NF449 is considered highly selective for the P2X1 receptor even at nanomolar concentrations^32,42^. Concentrations in the micromolar range, such as used by others^43,44^ and in the current study, have a higher P2X1 antagonist potency, but even a certain effect on other purinergic receptors^32^. P2X1 receptor silencing followed by caspase detection after challenging HeLa cells with Stx1 showed a protective effect similar to NF449 blockade. The effect of ATP blockade on calcium influx *in vitro*, and microvesicle release *in vitro* and *in vivo*, was also confirmed using the non-selective P2X antagonist, suramin, with a varying effect on all P2X receptors^32^.

Stx2 induces the release of microvesicles from blood cells^18^ and, as shown here, Stx1 and Stx2 induce the release of microvesicles from HeLa cells and platelets. Microvesicles contain cytosolic content from their parent cell, and, in the case of a toxin-affected cell, the microvesicles will carry toxin and thereby transfer their cargo to a recipient cell^16^. This mechanism of transfer of injurious substances via microvesicles will evade the host response as the toxin will be protected by host membranes. Thus, a substance capable of decreasing microvesicle release could be protective, in conditions in which microvesicles promote disease in general^45,46^, and in Stx-mediated disease in particular. NF449 inhibited Stx-induced microvesicle release from HeLa cells and platelets, and suramin reduced microvesicle release from platelets, both *in vitro* and *in vivo*. Calcium influx is required for microvesicle shedding^47^ and thus the decreased microvesicle release in the presence of NF449 and suramin would presumably be associated with blocked calcium influx via the P2X1 receptor^48^.

Suramin is used commercially for the treatment of parasite infections in humans^49^ as well as autistic spectrum disorders in mouse models^50^. In a recent study we reported that red blood cells stimulated with Stx2 released microvesicles, an effect modulated by the non-selective P2X receptor inhibitors suramin and PPADS^15^. We propose that the beneficial effects of NF449 and suramin on microvesicle release should be further explored as microvesicles have been associated with the transfer of deleterious substances in various disease states^46^.

We conclude that Stx induces an intracellular signal via ATP interaction with purinergic receptors allowing calcium influx and promoting the injurious effects of the toxin on cell viability, the inhibition of protein synthesis and apoptosis. Furthermore, the ATP-induced signal stimulates microvesicle release from cells. This may be a protective mechanism by which cells rid themselves of toxic substances but may also promote disease, as these microvesicles circulate with toxic content thus reaching the kidney which is the target organ^16^. We demonstrate that purinergic P2X blockade abrogated the injurious effects of Stx related to calcium influx, viability and microvesicle release and therefore suggest purinergic signaling as a novel mechanism of Stx-mediated cellular injury. Purinergic signaling blockade should be explored as a novel therapeutic option in Stx-mediated disease.

## Methods

### HeLa cells and platelets

HeLa cells were cultured in Dulbecco’s Modified Eagle Medium (DMEM, Invitrogen, Paisley, UK) with supplements and seeded at a density of 10^5^ cells/mL 24 hours before the start of experiments, as described in the supplement.

For the isolation of platelet-rich-plasma, blood was drawn from healthy adult donors into Vacutainer tubes (Becton Dickinson, Franklin Lanes, NJ) containing Lepirudin (50 μg/mL, Refludan, Celgene, Windsor, UK), as detailed in the supplement. The study was performed with the approval of the Regional Ethics Review Board of Lund University, the written informed consent of the subjects (healthy adult donors) in accordance with relevant guidelines and regulations.

### Detection of the P2X1 receptor on HeLa cells and platelets

The presence of the P2X1 receptor on HeLa cells and platelets was confirmed by immunoblotting (see description in the supplement) using an anti-P2X1 primary antibody (1 μg/mL, ab74058, Abcam, Cambridge, UK). The observed band corresponded to approximately 60 kDa, which is the receptor size reported for P2X1^35^, shown in Supplementary Fig. S6.

### P2X1 silencing

P2X1 mRNA in HeLa cells was silenced by RNA interference. Immunoblotting was performed to confirm protein reduction (Supplementary Fig. S7), described in the supplement.

### Shiga toxins

Stx1 and Stx2 were obtained from the Phoenix Lab (Phoenix Lab, Tufts Medical Center, Boston, MA). In certain experiments Alexa:488-conjugated Stx1B-subunit^51^ was used. For details see the supplement.

### Detection of ATP

Detection of ATP in mouse plasma and medium from HeLa cells was carried out using firefly luciferase by a bioluminescence assay, as described in the supplement.

### Phosphate determination assay

Free phosphate groups in media from HeLa cells stimulated with Stx1, PBS or calcium ionophore were detected using a Phosphate assay kit (Sigma-Aldrich, Saint Louis, MO), as detailed in the supplement.

### NF449

NF449 (Tocris Bioscience, Bristol, UK) was used as a specific P2X1 receptor antagonist^42^ at a concentration of 60 μM, unless otherwise stated. NF449 did not bind to Stx1 or Stx2 in the fluid phase, nor did it affect Stx binding to cells, its uptake or intracellular cleavage as further described in the supplement and Supplementary Fig. S8 (binding) and S9 (uptake).

### Suramin

Suramin (Sigma-Aldrich) is a non-selective P2X receptor antagonist^26^, which was used for *in vitro* and *in vivo* experiments described below.

### Calcium influx assay

Calcium concentrations in HeLa cells and platelet-rich-plasma were measured using Fluo-4 NW (Thermo Fisher Scientific), as described in the supplementary methods.

### Stx1B-subunit retrograde transport to the endoplasmic reticulum

Retrograde trafficking of Stx1B to the endoplasmic reticulum was detected by a previously described method^52^, using HeLa cells transfected with a SNAP-tag localized to the ER and benzylguanine-labeled Stx1B, as described in the supplement.

### Viability assay

The viability of HeLa cells exposed to Stx was assessed using Alamar Blue (Thermo Fisher Scientific). The viability of PBS-treated cells was defined as 100%. Details are given in the supplement.

### Protein synthesis assay

HeLa cell protein synthesis was assessed by [^35^S]-methionine protein incorporation. Values are presented as counts per minute from [^35^S] incorporated into newly synthesized protein divided by total protein concentration, as described in the supplement.

### Caspase 3/7 apoptosis assay

Apoptosis assays were carried out using CellEvent Caspase-3/7 reagent and NucBlue nuclear staining (both from Thermo Fisher Scientific). Fluorescence emitted from HeLa cells was measured and divided by the number of cell nuclei, see the supplement.

### Isolation and detection of microvesicles from HeLa cells and platelets

HeLa cells were stimulated with Stx1B:Alexa488 or Stx2. Microvesicles were isolated and labeled with mouse anti-CD44PE and rabbit anti-Stx2 (BEI resources, Manassas, VA) and swine anti-rabbit FITC (Dako, Glostrup, Denmark), both diluted in 0.1% saponin (Sigma-Aldrich). Platelets were stimulated with Stx1 or Stx2. Microvesicles were isolated and labeled with mouse anti-CD42PE and mouse anti-Stx1 (Santa Cruz Biotechnology, Dallas, TX) and goat anti-mouse FITC (Dako), both diluted in 0.1% saponin, or rabbit anti-Stx2 as above. The procedure is detailed in the supplement.

### Mice

BALB/c wild-type mice were bred in the animal facilities of the Biomedical Service Division, Medical Faculty, Lund. Both female and male mice were used at 8**–**13 weeks of age and were age-matched. All animal experiments were approved by the animal ethics committee of Lund University and carried out in accordance with the guidelines of the Swedish National Board of Agriculture and the EU directive for the protection of animals used in science. Approval numbers M13–14 and M148–16.

### Shiga toxin 2-injection mouse model

Stx2 was injected intraperitoneally at 285, 142.5 or 71.25 ng/kg body weight and control mice received PBS, as previously described^27^. Mice were monitored daily. In the Stx2-injection model mice usually show symptoms on day 3. For microvesicle counts mice were sacrificed on day 3 and for ATP assay mice were sacrificed upon showing signs of disease or on day 7. The procedure is detailed in the supplement.

### *Escherichia coli* O157:H7-infection mouse model

Mice were infected with *E. coli* O157:H7 (10^8^ CFU/mouse) as previously described^27,53^. Mice were sacrificed on day 3 after inoculation, before the development of symptoms. Blood was collected for microvesicle analysis as described above. See the supplement for details.

### Treatment of Stx2-injected and EHEC-infected mice with suramin

BALB/c mice were injected intraperitoneally with suramin and control mice received vehicle 16 hours before injection of Stx2 intraperitoneally or one hour before inoculation with *E. coli* O157:H7 or its corresponding vehicle. All mice were sacrificed on day 3 post inoculation and samples were collected for microvesicle analysis, as described in the supplement.

### Isolation and labeling of murine microvesicles

Microvesicles were isolated and labeled as previously described^16^. Platelet microvesicles were detected with rat anti-mouse CD41:APC (BD Biosciences) and Stx2-containing microvesicles were detected as described above. See the supplement for details.

### Flow cytometry for detection of cells and microvesicles

Cells were analyzed using a FACSCanto^TM^II flow cytometer (BD Immunocytometry Systems, San Jose, CA) and microvesicles were analyzed using a CyFlow Cube 8 (Sysmex Partec, Görlitz, Germany), as described in the supplement.

### Statistical analysis

Differences between groups were assessed by the two-tailed Mann-Whitney U test, or by the Kruskal-Wallis multiple-comparison test when comparing more than two groups, followed by comparison between specific groups using the Dunn procedure. For calcium influx repeated measurements two-way repeated measures ANOVA was used. All statistical analyses were calculated using Prism 7 version 7.0a (GraphPad, La Jolla, CA).

## Supplementary information

Supplementary Information

## Data Availability

Data are described in the supplement and available from the corresponding author upon request.
