# Supplementary material for: Shiga toxin signals via ATP and its effect is blocked by purinergic receptor antagonism
Source: Sci Rep. 2019 Oct 7;9:14362. doi: 10.1038/s41598-019-50692-1 (PMC6779916; doi:10.1038/s41598-019-50692-1)
Supplement: Supplementary file 1 — Supplementary Information [file 41598_2019_50692_MOESM1_ESM.pdf]

Shiga toxin signals via ATP and its effect is blocked by purinergic receptor antagonism

Karl E. Johansson, Anne-Lie Ståhl, Ida Arvidsson, Sebastian Loos, Ashmita Tontanahal, Johan Rebetz, Milan Chromek, Ann-Charlotte Kristoffersson, Ludger Johannes, Diana Karpman

Supplementary information

Complete methods

HeLa cells and platelets

HeLa cells were cultured in Dulbecco's Modified Eagle Medium (DMEM, Invitrogen, Paisley, UK) supplemented with 10% fetal bovine serum and 1% penicillin-streptomycin. Cells were cultured in a cell incubator with 5 % CO_2 in 37°C. HeLa cells were seeded at a density of 10^5 cells/mL 24 hours before the start of experiments.

For the isolation of platelet-rich-plasma, blood was drawn from healthy adult donors into Vacutainer tubes (Becton Dickinson, Franklin Lanes, NJ) containing Lepirudin (50 µg/mL, Refludan, Celgene, Windsor, UK) via venipuncture, using a butterfly needle (Terumo Medical products, Hangzhou, China). The first blood tube (2.7 ml) was discarded. Whole blood was centrifuged at 170 x g for 15 min and the supernatant, containing platelet-rich-plasma, was collected. The study was performed with the approval of the Regional Ethics Review Board of Lund University and the written informed consent of the subjects (healthy adult donors) and in accordance with the relevant guidelines and regulations.

Detection of the P2X1 receptor on HeLa cells and platelets

The presence of the P2X1 receptor on HeLa cells and platelets was confirmed by immunoblotting. Cells were lysed in RIPA lysis buffer (0.15 M NaCl, 30 mM HEPES, 1% Triton X-100, 1% sodium deoxycholate and 0.1% sodium dodecyl sulfate). Whole cell lysate proteins were reduced with 2-mercaptoethanol (Sigma-Aldrich, Saint Louis, MO), separated by SDS-PAGE and transferred to a nitrocellulose membrane. The P2X1 receptor was stained with anti-P2X1 primary antibody (1 μg/mL, ab74058, Abcam, Cambridge, UK) followed by anti-rabbit HRP as the secondary antibody (0.5 μg/mL, ab7171, Abcam). P2X1 is shown in Supplementary Figure S6. The size corresponds to approximately 60 kDa, as previously described.[1]

P2X1 silencing

P2X1 mRNA in HeLa cells was silenced by RNA interference. Cells were transfected with a pool of three different siRNAs targeting P2X1 mRNA (siP2X1, 3 and 6 μM) or with a non-targeting control siRNA (siCtrl, 6 μM) using siRNA Transfection Reagent according to the manufacturer's instructions (Santa Cruz Biotechnology, Santa Cruz, CA). Experiments were carried out 48 h post-transfection. Immunoblotting was performed to confirm protein reduction (Supplementary Figure S7). Equal loading was determined by the stain-free method (BioRad, Hercules, CA). These cells were used for Caspase 3/7-detection, as described below.

Shiga toxins

Stx1 and Stx2 were obtained from the Phoenix Lab (Phoenix Lab, Tufts Medical Center, Boston, MA). LPS contamination was measured using the Limulus Amebocyte Lysate

method (Thermo Fisher Scientific, Rockford, IL) detecting small amounts (in Stx1 2.3 ng/mg toxin, in Stx2 183 ng/mg toxin). In certain experiments Alexa488-conjugated Stx1B-subunit[2] was used.

Detection of ATP

Detection of ATP was carried out using a bioluminescence assay. Mouse plasma was diluted 1:1000 in PBS and ATP content was analyzed using firefly luciferase to cleave phosphate groups (65 nM, Sigma-Aldrich) and D-luciferin (1.3 mM, Thermo Fisher Scientific) with detection at one-sec integration time in a Glomax Discover System (Promega, Fitchburg, WI). HeLa cells were incubated with Hank's Balanced Salt Solution with Ca^{2+}/Mg^{2+} (HBSS, GE Life Sciences, Chicago, IL) supplemented with 20 mM HEPES for 5 min at 37°C followed by addition of Stx1 (1 µg/mL or 200 ng/mL) or Stx2 (1µg/mL). PBS (GE Life Sciences) was the negative control and histamine (100 µM, Sigma-Aldrich) was the positive control. After 5 min the cell medium was collected and added to 20x reaction buffer. The ATP concentration in the medium was measured using ATP determination kit (Thermo Fischer Scientific) according to the manufacturer's instructions.

Phosphate determination assay

HeLa cells were incubated with TRIS-buffered saline (Medicago, Uppsala, Sweden) supplemented with 1 mM $CaCl_2$, 0.4 mM $MgSO_4$ and 20 mM HEPES for 5 min at 37°C before addition of Stx1 (1 µg/mL), A23187 calcium ionophore as the positive control (10 µM, Sigma-Aldrich) or PBS. Supernatant was collected after 40 min and phosphate reagent (Phosphate assay kit, Abcam) was added to each sample and to the control (blank) samples, containing PBS that had not been in contact with the cells, according to

the manufacturer's instructions. After 30 min incubation at room temperature the absorbance of the samples was measured at 600 nm and the blank value was subtracted from each sample.

NF449

NF449 (Tocris Bioscience, Bristol, UK) is a specific P2X1 receptor antagonist.[3] NF449 was used at a concentration of 60 µM, unless otherwise stated.

In order to rule out a direct interaction between NF449 and Stx microtiter wells were coated with NF449 (120 µM) in 0.1 M $NaHCO_3$ pH 9.3 (Merck, Darmstadt, Germany) at 4°C overnight. Wells were washed with PBS-Tween 0.05% (PBS-T, Medicago, Uppsala, Sweden) and blocked with bovine serum albumin (1%, BSA, Sigma-Aldrich) for 1 h. Wells were washed and incubated with Stx1 or Stx2 (1 µg/mL in 1% BSA) for 1 h. After washing, wells were incubated with rabbit anti-Stx1 antibody (1:1000, List Labs, Campbell, CA) or rabbit anti-Stx2 (1:1000, BEI resources, Manassas, VA) for 1 h, washed and further incubated with anti-rabbit HRP antibody (1:1000, Dako, Glostrup, Denmark) for 1 hour. Microtiter wells were developed with SuperSignal ELISA Pico Chemiluminescent Substrate and luminescence was detected in a GloMax Discover System. No Stx binding could be detected, suggesting that there was no direct interaction between NF449 and Stx under these conditions.

Experiments were further carried out to determine if NF449 affected Stx1 binding to cells. HeLa cells were incubated with NF449 in PBS, or PBS alone, at 37°C for 1 h followed by incubation with Stx1B-subunit:Alexa 488 (1 µg/mL), for 15 min, on ice to prevent toxin uptake. Cells were washed and detached with ice-cold EDTA (Versene,

Thermo Fisher Scientific) and analyzed by flow cytometry (described below). No difference in fluorescence could be detected by flow cytometry, indicating that NF449 did not affect Stx1B-binding (Supplementary Figure S8).

Experiments were designed to examine an effect of NF449 on toxin cellular uptake. HeLa cells were treated with NF449 or PBS for 30 min followed by incubation with Stx1 (1000, 200, 7 or 0 ng/mL) for 4 h at 37°C. Cells were washed with PBS and lysed in RIPA buffer. Cell lysate was transferred to white Maxisorp 96-well plates that were coated with mouse anti-Stx1 antibodies (2 µg/mL, STX1-3C10, Toxin Technology, Sarasota, FL) and blocked with 1 % BSA. The cell lysate was incubated for 1 h followed by detection with rabbit anti-Stx1 (1 µg/mL, List Biological Laboratories, Campbell, CA), goat anti-rabbit HRP (1:1000) and SuperSignal ELISA Pico Chemiluminescent Substrate (Thermo Fisher Scientific). Luminescence was detected in a GloMax Discover System. No difference between untreated and NF449-treated samples could be detected with regard to Stx1 uptake (Supplementary Figure S9).

Stx1 A-subunit has a molecular weight of 32 kDa and its intracellular cleavage product, A_1, is expected to have a molecular weight of 27.5 kDa.[4] To investigate whether NF449 had an effect on cleavage of the Stx1 A-subunit, Stx1 was labeled with Iodine-125 (^{125}I) using Chloramine T (1 mg/mL) as an oxidant. HeLa cells were incubated with NF449 or with PBS for 30 minutes before addition of Stx1 (7 ng/mL) together with a trace amount of Stx1-^{125}I for 4 hours. The cells were lysed in RIPA buffer before separation by SDS-PAGE and transferred to a nitrocellulose membrane. The membrane was placed in an intensifying screen (Dupont Cronex Lightning Plus LE) with an X-ray film (Amersham Hyperfilm ECL, GE Healthcare) for three days at -80°C before development. In the

presence or absence of NF449 there was no difference in the ratio between uncleaved and cleaved Stx1A.

Suramin

Suramin (Sigma-Aldrich) is a non-selective P2X receptor antagonist,[5] which was used for *in vitro* and *in vivo* experiments, described below.

Calcium influx assay

HeLa cells were incubated with Fluo-4 NW (Thermo Fisher Scientific) according to the manufacturer's instructions for 30 min at room temperature. In certain experiments NF449 or suramin (200 μM), was added directly into the Fluo-4 NW solution at the same time point. HeLa cells were placed in an Axio Observer.A1 microscope (Zeiss, Oberkochen, Germany) at 37°C and the cells were allowed to settle for 15 min before the start of the experiment. Each well was monitored for a total of 300 sec and images were taken at 30-sec intervals. Stx1 (1 μg/mL, this concentration was previously shown to induce calcium influx[6]), HBSS, as the negative control, or ATP (3 μM), as the positive control, were added to the wells 30 sec after the start of the experiment and A23187 (10 μM), was added after an additional 270 sec to induce fulminant calcium influx. Background signal was subtracted from the mean fluorescence intensity change of all cells in the field of view using ImageJ software (Version 1.48v, NIH, Bethesda).

Human platelet-rich-plasma was incubated with Fluo-4 NW, as above, and placed in a black 96-well plate with clear bottoms (Corning Inc., Corning, NY) in a GloMax Discover System at 37°C and further simultaneous stimulated with Stx1 or Stx2 (1 μg/mL) and LPS from *E. coli* O157 (1 μg/mL, a gift from R. Johnson, Public Health

Agency, Guelph, ON, Canada), with LPS alone or with PBS. LPS was added in order to activate the platelets and enable Stx binding, as previously shown.[7,8] In experiments in which Stx2 was used apyrase (0.32 U/mL, Sigma-Aldrich) was added to enzymatically remove previously present ATP and reduce desensitization.[9] Certain wells were pre-incubated with NF449. Initial fluorescence values (at 475 nm and 525/25 nm excitation and emission filters) were subtracted from 2 min post-stimulation values.

Stx1B-subunit retrograde transport to the endoplasmic reticulum

Retrograde trafficking of Stx1B to the ER was detected using a previously described method[10] in which the cell is transfected with a SNAP-tag targeting the ER that covalently binds to benzylguanine conjugated to Stx1. The SNAP-tag transfected HeLa cells were treated with NF449, PBS or the intracellular calcium chelator BAPTA-AM as a control (10 µM, Thermo Fisher Scientific) for 30 min, followed by incubation with Stx1B-subunit (1 µg/mL) tagged with O^6-benzylguanine (New England BioLabs, Ipswish, MA) on ice for an additional 30 min. The cells were washed three times with complete DMEM and incubated for 2 h at 37°C. SNAP-Cell Block (10 µM, New England BioLabs) was added for 30 min. Cells were washed 3 times with PBS and lysed in RIPA-buffer on ice for 30 min. The lysate was added to a 96-well ELISA plate (Nunc, Roskilde, Denmark) that had been pre-coated with anti-SNAP antibody (A00684-40, GenScript, Biotech, Piscataway, NJ) diluted 1:2500 in $NaCHO_3$ (50mM, pH 9.6), blocked with 0.2 % bovine serum albumin (Sigma-Aldrich) followed by anti-Stx1 antibody (2 µg/mL, STX1-3C10), secondary antibody anti-mouse biotinylated IgG (1:15000, Abcam) and streptavidin-peroxidase (1:5000, Sigma-Aldrich). Wells were developed with TMB Blue Substrate Chromogen (Agilent Technologies, Santa Clara, CA), acidified with sulfuric acid and absorbance was measured at 450 nm.

Viability assay

HeLa cells were incubated with Stx1 or Stx2 (7 ng/mL) or PBS control for 24 h in serum-free DMEM. Certain wells were pretreated with NF449 1 h before the toxin was added. Cell viability was assessed with Alamar Blue (Thermo Fisher Scientific) according to the manufacturer's instructions and analyzed using a GloMax Discover System. PBS-treated cells were defined as 100% viability.

Protein synthesis assay

HeLa cells were treated with NF449 or PBS for 30 min, followed by incubation with Stx1 (7 ng/mL) for 4 h. The cells were washed three times with PBS and incubated with [^{35}S]-labeled methionine diluted in methionine-free RPMI 1640 medium for 2 h. Cells were washed and lysed in RIPA-buffer. Full protein content of the lysates was precipitated in 14 % trichloroacetic acid followed by a wash in ice-cold acetone. Samples were diluted in scintillation liquid (Beckman Coulter, Brea, CA) and β-radiation was counted in a scintillation counter (LS 6500 Multi-Purpose Scintillation Counter, Beckman Coulter). Values are presented as counts per minute from [^{35}S] incorporated into newly synthesized protein divided by total protein concentration.

Caspase 3/7 apoptosis assay

HeLa cells were co-incubated with Stx1 (7 ng/mL) or the PBS vehicle and CellEvent Caspase-3/7 reagent (5 μM, Thermo Fisher Scientific). Certain wells were pre-treated with NF449 1 h before the toxin was added. After 24 h the cells were washed and incubated with HBSS containing 1 μg/mL NucBlue nuclear stain (Thermo Fisher Scientific) for 15 min. Cells were imaged in an Axio Observer.A1 microscope and the

fluorescence emitted with a 460 – 490 nm filter was measured and divided by the number of cell nuclei using ImageJ.

Isolation and detection of microvesicles from HeLa cells and platelets

HeLa cells were incubated in DMEM (Gibco, Carlsbad, CA) complemented with 0.1 % exosome-free fetal calf serum (Gibco) with or without NF449 for 1 h. Stx1B:Alexa488 (130 ng/mL) or Stx2 (200 ng/mL, both corresponding to 3 nM, a concentration that has previously been shown to cause microvesicle release from blood cells[11]) in PBS, or PBS alone, was added to the cells and incubated for 40 min. Microvesicle-containing supernatant was collected and centrifuged at 300g for 10 min followed by centrifugation at 10000g for 15 min to remove cells and cellular debris.

For isolation of platelet microvesicles whole blood was diluted 1:1 with DMEM, incubated with NF449 or suramin (200 μM) and stimulated with Stx1 or Stx2 (both at 200 ng/mL) or with PBS. Blood cells were removed by centrifugation at 2600g for 10 min and small platelets and cellular debris was removed by centrifugation at 10000g for 15 min. Microvesicles were fixed in 1% paraformaldehyde (PFA, HistoLab, Gothenburg, Sweden) for 30 min.

HeLa cell microvesicles were labeled with mouse anti-human CD44PE (1:400, eBioscience, Thermo Fisher Scientific) and platelets microvesicles were labeled with mouse anti-human CD42PE (1:80, Dako). For detection of Stx1 mouse anti-Stx1 (1.25 μg/mL, Santa Cruz Biotechnology, Dallas, TX) and goat anti-mouse FITC (1:1000, Dako), both diluted in 0.1% saponin (Sigma-Aldrich), were used. Stx2 was detected using rabbit anti-Stx2 (1:200) and swine anti-rabbit FITC (1:2000, Dako), both diluted in

0.1% saponin. All antibody incubation times were 30 min. Microvesicles were washed with PBS and centrifuged at 20000g for 40 min and the pellet was saved. Buffers, cell medium and PFA were filtered through a 0.2 µm pore-size filter (Pall Corporation, Ann Arbor, MI) to reduce aggregates.

Mice

BALB/c wild-type mice were bred in the animal facilities of the Biomedical Service Division, Medical Faculty, Lund. Both female and male mice were used at 8–13 weeks of age and were age-matched. All animal experiments were approved by the animal ethics committee of Lund University in accordance to the guidelines of the Swedish National Board of Agriculture and the EU directive for the protection of animals used in science. Approval number M13-14 and M148-16.

Shiga toxin 2-injection mouse model

Purified Stx2 was diluted in PBS and injected intraperitoneally at a dose of 285, 142.5 or 71.25 ng/kg body weight for ATP assay and 285 ng/kg for microvesicle analysis and control mice received PBS vehicle at the same volume, as previously described.[12] Mice were monitored two to four times a day. Weight was taken daily and mice were observed for signs of disease (ruffled fur, lethargy, hunched posture, decreased activity, paralysis, tremor, ataxia and weight loss ≥ 20%), as previously described.[12] In the Stx2-injection model mice usually show symptoms from day 3 onwards. For microvesicle counts mice were sacrificed on day 3 and for ATP assay mice were sacrificed upon showing signs of symptoms or on day 7 (the defined end of the experiment). After isoflurane anesthesia blood was collected by heart puncture into citrated syringes, and the animals were sacrificed by cervical dislocation. For analysis of microvesicles blood was treated with

sterile-filtered PFA 4% (Histolab) at a final concentration of 2%.

***E. coli* O157:H7**

The Stx2-producing *E. coli* O157:H7 strain 86–24 (kindly provided by A. D. O'Brien, Uniformed Services University of the Health Sciences, Bethseda, MD) was previously characterized.[13] Bacteria were grown, centrifuged and resuspended to a concentration of 10^9 colony forming units (CFU)/mL, as previously described.[12] Each mouse was inoculated with 10^8 CFU in a volume of 100 µl.

***Escherichia coli* O157:H7-infection mouse model**

Mice were infected with *E. coli* O157:H7 according to a previously described infection protocol.[12] Mice were monitored two to four times a day. Weight was taken daily and mice were observed for signs of disease as previously described.[12] In this mouse model mice usually develop symptoms on day 6 and onwards. Mice were sacrificed on day 3 after inoculation, before the development of symptoms, to obtain microvesicle levels at this specific time-point. Before sacrifice mice were anesthetized with isoflurane, blood was collected for microvesicle analysis as described above. All mice were tested for bacteremia using blood culture flasks (Biomérieux inc, Durham, NC) and found to be negative.

Treatment of Stx2-injected and EHEC-infected mice with Suramin

BALB/c mice were injected intraperitoneally with suramin diluted in NaCl 0.9% at a dose of 60 mg/kg bodyweight and control mice received NaCl 0.9 % (vehicle) at the same volume 16 hours before injection of Stx2 intraperitoneally or with a suramin dose of 20 mg/kg body weight, or corresponding vehicle, one hour before inoculation with *E.*

coli O157:H7. All mice were sacrificed on day 3 post inoculation and samples were collected for microvesicle analysis.

Isolation and labeling of murine microvesicles

Microvesicles were isolated and labeled as previously described.[11] Briefly, whole blood from mice fixed in PFA was centrifuged to isolate microvesicles and to obtain a microvesicle-enriched suspension. Microvesicles from platelets were detected with rat anti-mouse CD41:APC (1:40, platelet marker) or IgG1:APC as an isotype control (both antibodies from BD Biosciences) and Stx-containing microvesicles were detected with polyclonal rabbit anti-Stx2 (1:200) and swine anti-rabbit:FITC (1:300, Dako, Glostrup, Denmark), both diluted in 0.1% saponin (Sigma-Aldrich).

Flow cytometry for detection of cells and microvesicles

FACSCanto™II flow cytometer with a 488 nm laser and FACSDiva V.6.0 (BD Immunocytometry Systems, San Jose, CA) was used for flow cytometry measurements of cells. Forward scatter (FSC) and side scatter (SSC) channels were recorded with linear gain. The fluorescence channel was recorded at logarithmic gain and the flow rate was set to medium. Ten thousand events were counted in the population gate for each sample. Results are presented as percentage of Stx1B:Alexa488-positive cells and units of mean fluorescent intensity.

Flow cytometry for detection of microvesicles was performed using a CyFlow Cube 8 flow cytometer equipped with a 488 nm and a 638 nm laser (Sysmex Partec, Görlitz, Germany) and True Absolute Volumetric Counting (TVAC). Intuitive CyFlow™ acquisition and analysis software packages (Sysmex Partec) were used. The acquired data

files were analyzed using FCS Express 4 Flow Research Edition software (Version 4.07.0003, De Novo Software, Glendale, CA). Sheath fluid was pre-filtered (0.2 μm filter) and de-gassed before use. FSC and SSC and fluorescence channels were recorded in logarithmic mode. The microvesicle gate was set using Megamix beads (BioCytex, Marseille, France) to determine upper limits in both FSC (200) and SSC (240) signals and the lower limits were placed above the background level of the instrument and/or buffer which was determined by running 0.2 μm-filtered PBS. The detection threshold was set at a determined FSC. The spectral overlap of the fluorochromes used in this assay was compensated for using single-stained controls for each fluorochrome. A microvesicle was defined as an event positive for a specific cell marker and <1 μm in size. Due to the detection limit of the flow cytometer particles below 100 nm in size are not detected. Results are presented as positive microvesicles after subtraction of the control antibody. A volume of 15 μl was counted for each sample on low flow rate (0.2 μl/s).

Statistical analysis

Differences between groups were assessed by the two-tailed Mann-Whitney U test, or by the Kruskal-Wallis multiple-comparison test when comparing more than two groups, followed by comparison between specific groups using the Dunn procedure. For calcium influx repeated measurements two-way repeated measures ANOVA was used. All statistical analyses were calculated using Prism 7 version 7.0a (GraphPad, La Jolla, CA).

References

1 Clifford, E. E., Parker, K., Humphreys, B. D., Kertesy, S. B. & Dubyak, G. R. The P2X1 receptor, an adenosine triphosphate-gated cation channel, is expressed in human platelets but not in human blood leukocytes. *Blood* **91**, 3172-3181, (1998).

2 Renard, H. F. *et al.* Endophilin-A2 functions in membrane scission in clathrin-independent endocytosis. *Nature.* **517**, 493-496, (2015).

3 Kassack, M. U. *et al.* Structure-activity relationships of analogues of NF449 confirm NF449 as the most potent and selective known P2X1 receptor antagonist. *Eur J Med Chem* **39**, 345-357, (2004).

4 Garred, O., van Deurs, B. & Sandvig, K. Furin-induced cleavage and activation of Shiga toxin. *J Biol Chem* **270**, 10817-10821, (1995).

5 Lambertucci, C. *et al.* Medicinal chemistry of P2X receptors: agonists and orthosteric antagonists. *Curr Med Chem* **22**, 915-928, (2015).

6 Klokk, T. I., Kavaliauskiene, S. & Sandvig, K. Cross-linking of glycosphingolipids at the plasma membrane: consequences for intracellular signaling and traffic. *Cell Mol Life Sci.* **73**, 1301-1316, (2016).

7 Ståhl, A. L., Sartz, L., Nelsson, A., Békássy, Z. D. & Karpman, D. Shiga toxin and lipopolysaccharide induce platelet-leukocyte aggregates and tissue factor release, a thrombotic mechanism in hemolytic uremic syndrome. *PLoS One.* **4**, e6990, (2009).

8 Ghosh, S. A., Polanowska-Grabowska, R. K., Fujii, J., Obrig, T. & Gear, A. R. Shiga toxin binds to activated platelets. *J Thromb Haemost* **2**, 499-506, (2004).

9 Rolf, M. G., Brearley, C. A. & Mahaut-Smith, M. P. Platelet shape change evoked by selective activation of P2X1 purinoceptors with alpha,beta-methylene ATP. *Thromb Haemost* **85**, 303-308, (2001).

10 Geiger, R., Luisoni, S., Johnsson, K., Greber, U. F. & Helenius, A. Investigating endocytic pathways to the endoplasmic reticulum and to the cytosol using SNAP-trap. *Traffic* **14**, 36-46, (2013).

11 Ståhl, A. L. *et al.* A novel mechanism of bacterial toxin transfer within host blood cell-derived microvesicles. *PLoS Pathog.* **11**, e1004619, (2015).

12 Calderon Toledo, C. *et al.* Shiga toxin-mediated disease in MyD88-deficient mice infected with Escherichia coli O157:H7. *Am J Pathol* **173**, 1428-1439, (2008).

13 Bekassy, Z. D. *et al.* Intestinal damage in enterohemorrhagic Escherichia coli infection. *Pediatr Nephrol* **26**, 2059-2071, (2011).

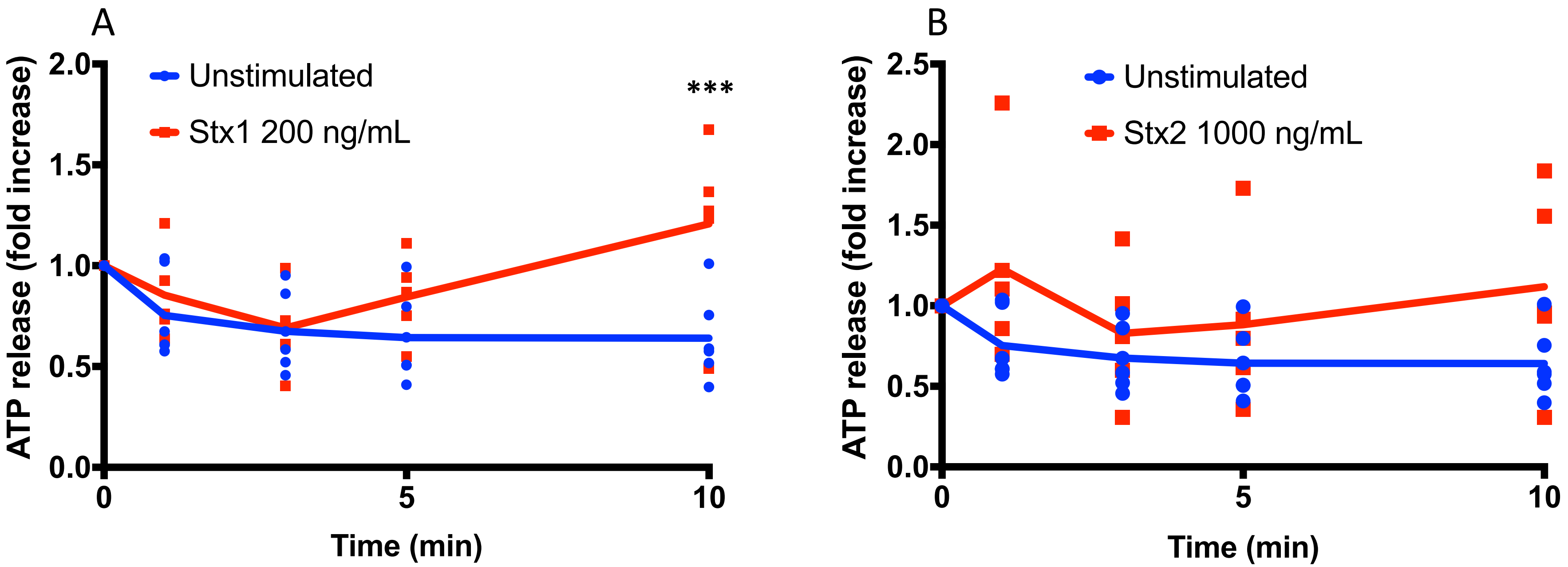

Supplementary Figure S1: Shiga toxin induces release of ATP *in vitro*

HeLa cells were stimulated with PBS (n=6), Shiga toxin 1 (Stx1, 200 ng/mL, n=5) (A) or Shiga toxin 2 (Stx2, 1000 ng/mL, n=5) (B) and the ATP content was measured after 1, 3, 5 and 10 min. Data is presented as fold difference of initial ATP value. The median extracellular ATP content is depicted as the colored curve. ***: P<0.001, two-way repeated measures ANOVA.

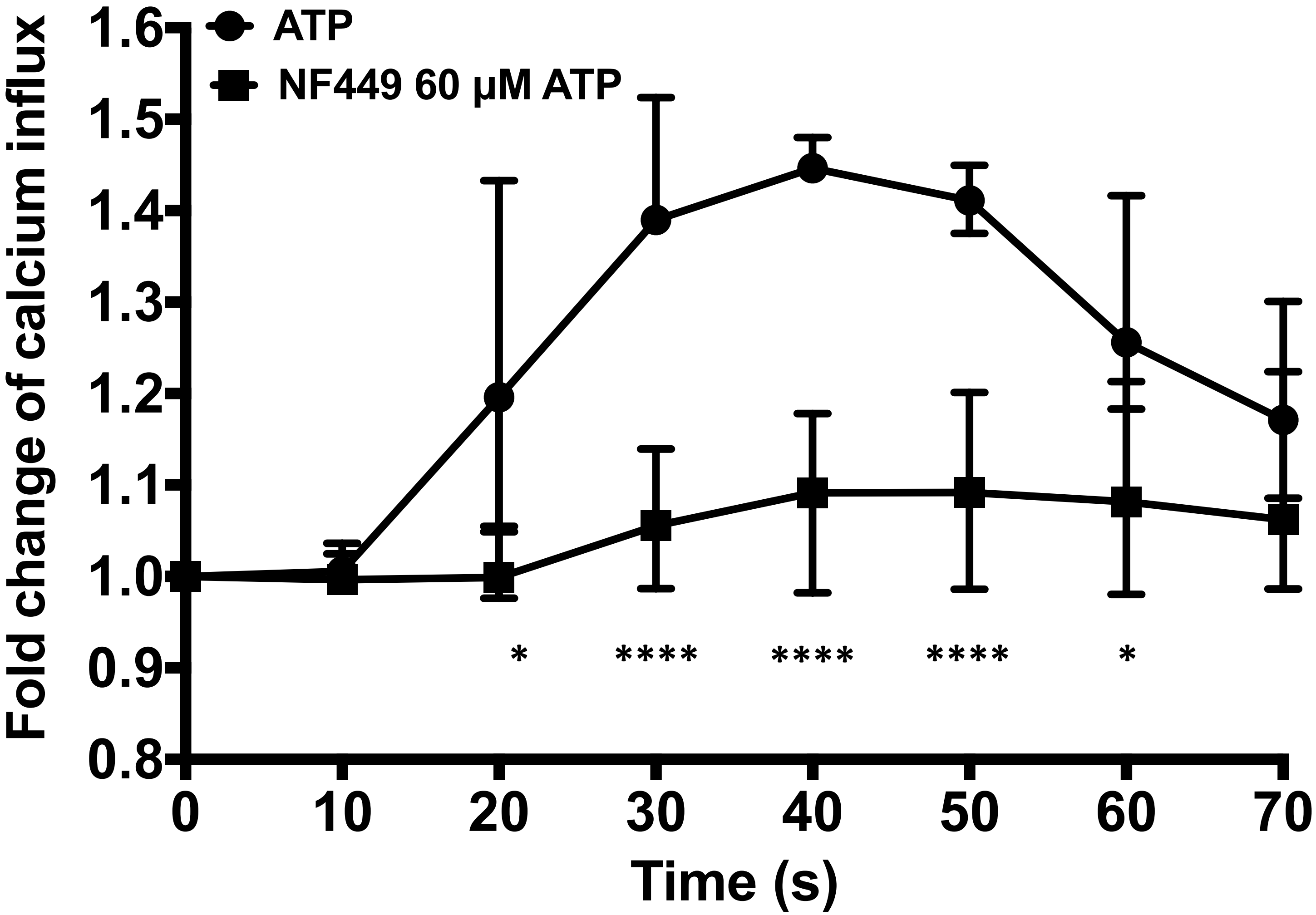

Figure S2: The effect of NF449 on calcium influx induced by ATP in HeLa cells

Calcium influx was measured in HeLa cells preincubated with NF449 (n=4) or PBS vehicle (n=3), stimulated with ATP (3 μM) and imaged by fluorescence microscopy. Results are shown as mean fluorescent change of all cells in the field of view and presented as median and range. *: $P<0.05$, ****: $P<0.0001$, two-way repeated measures ANOVA.

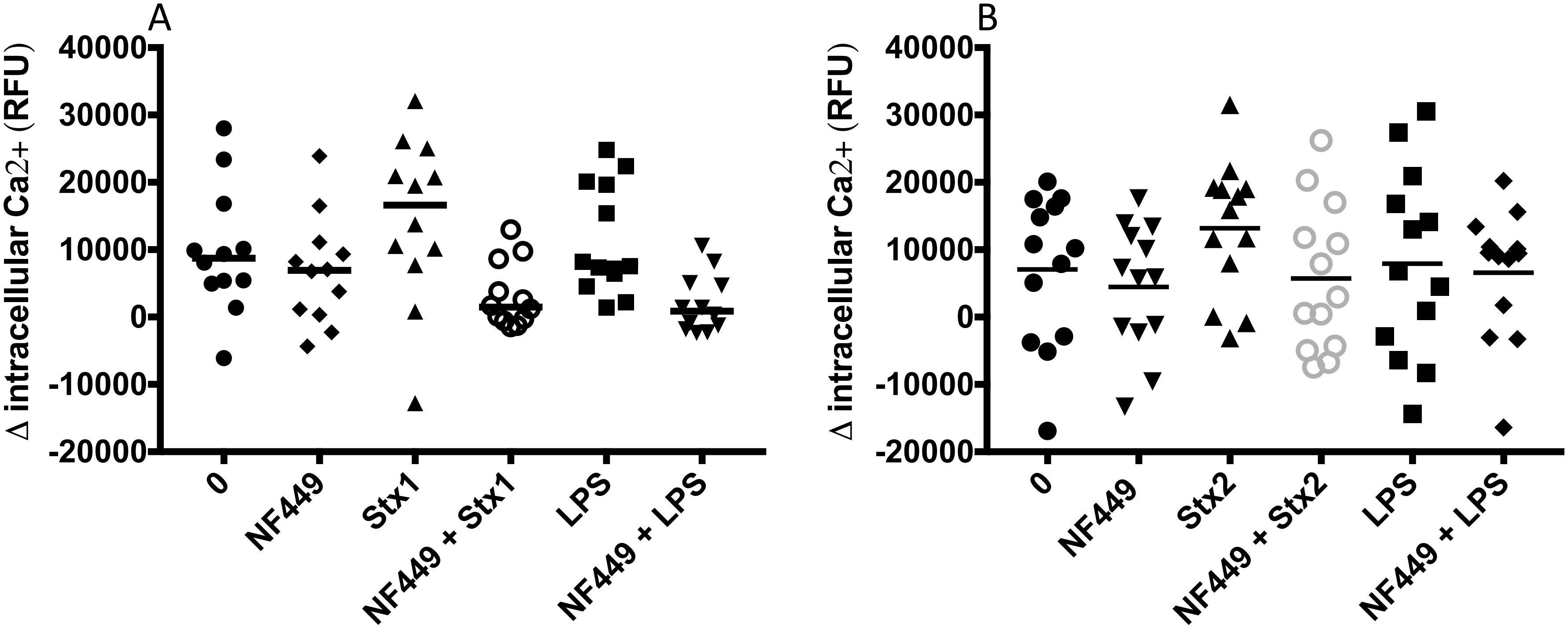

Figure S3: The effect of NF449 on calcium influx induced by Shiga toxin in platelets

Platelets (n=3 donors) were preincubated with NF449 or PBS vehicle followed by O157LPS, Stx1 (A) or Stx2 (B) or PBS vehicle. Data points for PBS-treated and NF449-treated platelets are the same as in Figure 2B and 2C. Data is presented as the initial fluorescence subtracted from fluorescence after 2 minutes. The median is denoted by the bar. RFU: relative fluorescent units.

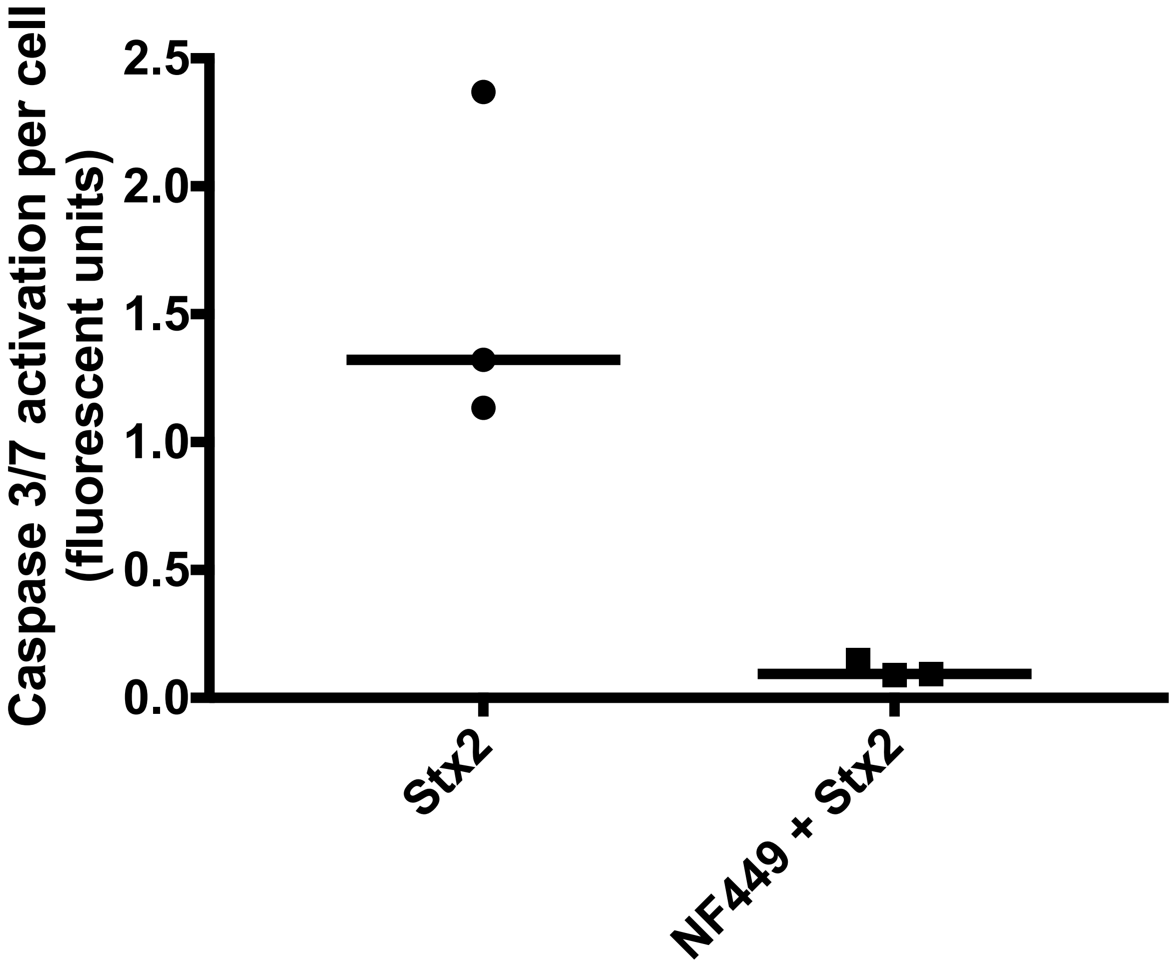

Figure S4: P2X1 blockade inhibits Stx2-induced apoptosis on HeLa cells

Shiga toxin 2 (Stx2)-induced caspase 3/7 activation was measured in HeLa cells pretreated with NF449 or left untreated, showing less caspase 3/7 activation in the cells that were pretreated with NF449. Data is presented as median caspase 3/7 activation per cell. The median is denoted by the bar.

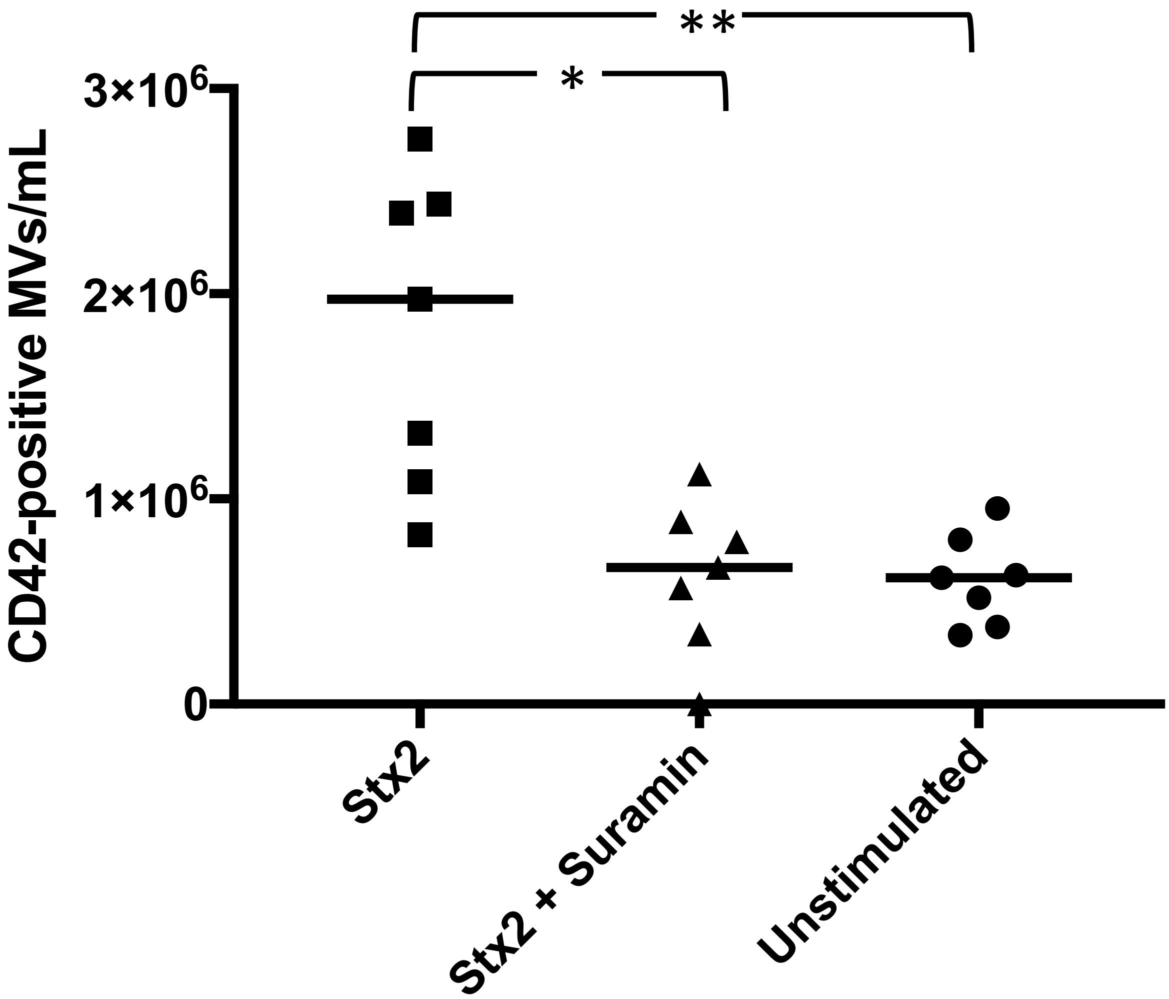

Figure S5: Suramin inhibits the release of platelet-derived microvesicles

Whole blood was pretreated with suramin or left untreated and stimulated with Shiga toxin 2 (Stx2). Stx2 induced a significant release of platelet-derived (CD42) microvesicles (MVs) that was reduced by suramin (median MVs in the unstimulated control was 6.2×10^5/mL). Data is presented as median MVs/mL. The median is denoted by the bar. *: P<0.05, **: P<0.01, Kruskal-Wallis test.

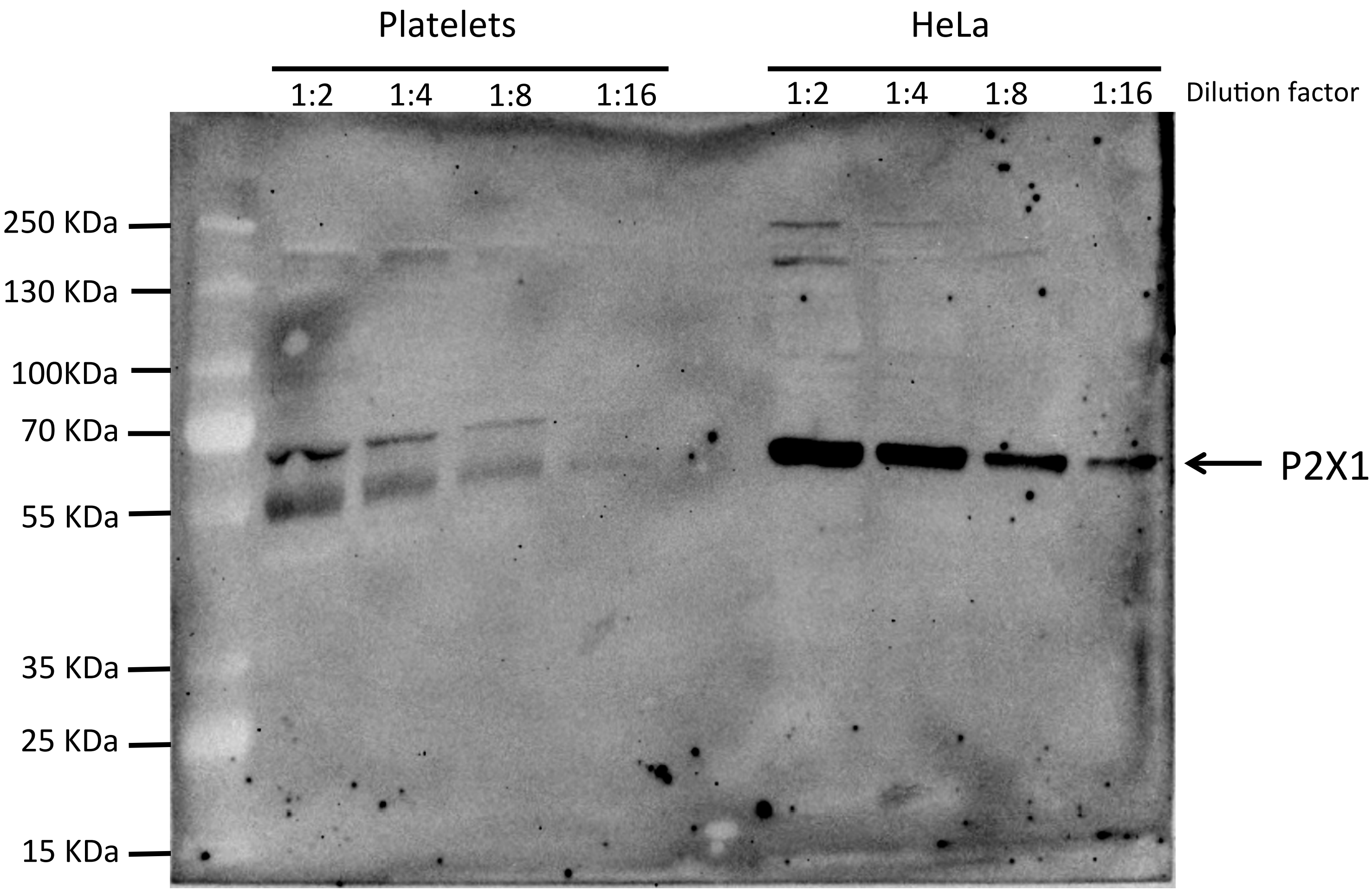

Figure S6: P2X1R in HeLa cells and platelets

HeLa cell and platelet lysates were separated by gel electrophoresis, transferred to nitrocellulose membranes and stained with an anti-P2X1 receptor antibody. Bands were detected by immunoblotting corresponding to approximately 60 kDa which is the size reported for P2X1.

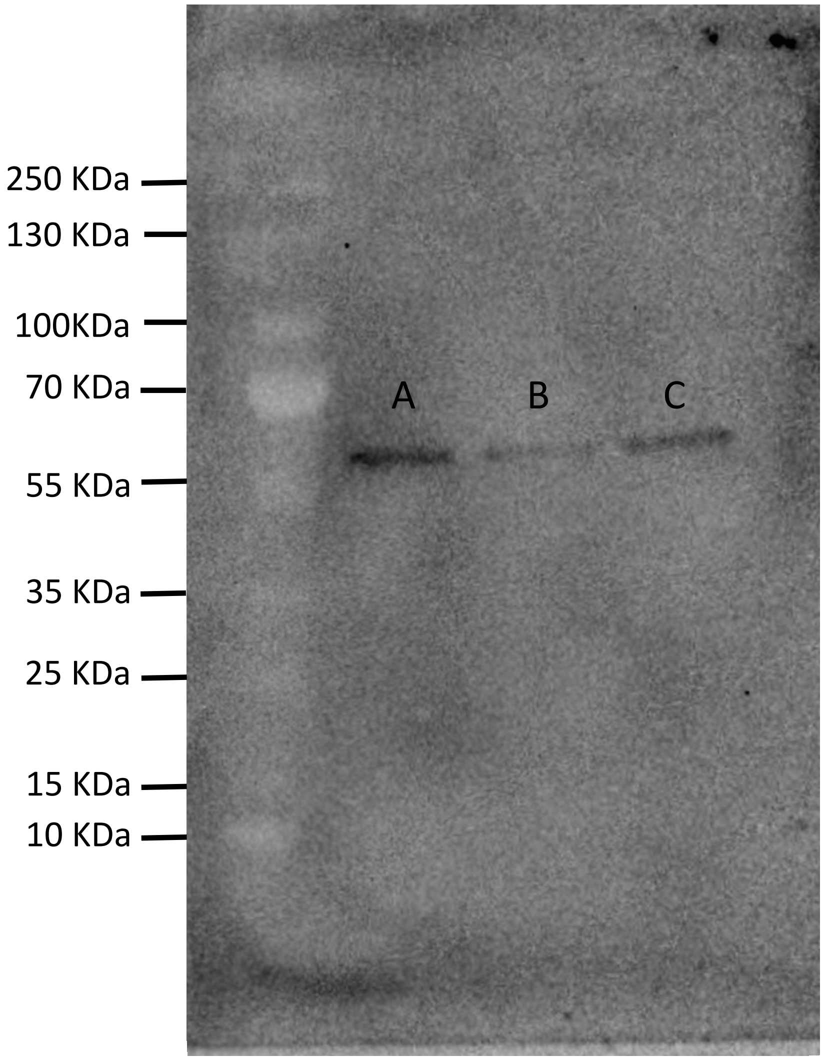

Figure S7: Silencing of P2X1 in HeLa cells

HeLa cells treated with (A) non-targeting control siRNA (siCtrl, 6 μM) (B) siRNA targeting P2X1 mRNA (siP2X1, 6 μM) or (C) siP2X1 (3 μM) were lysed and protein content was separated by electrophoresis. P2X1 content was detected by immunoblotting as before. Bands were detected at approximately 60 kDa, corresponding to P2X1.

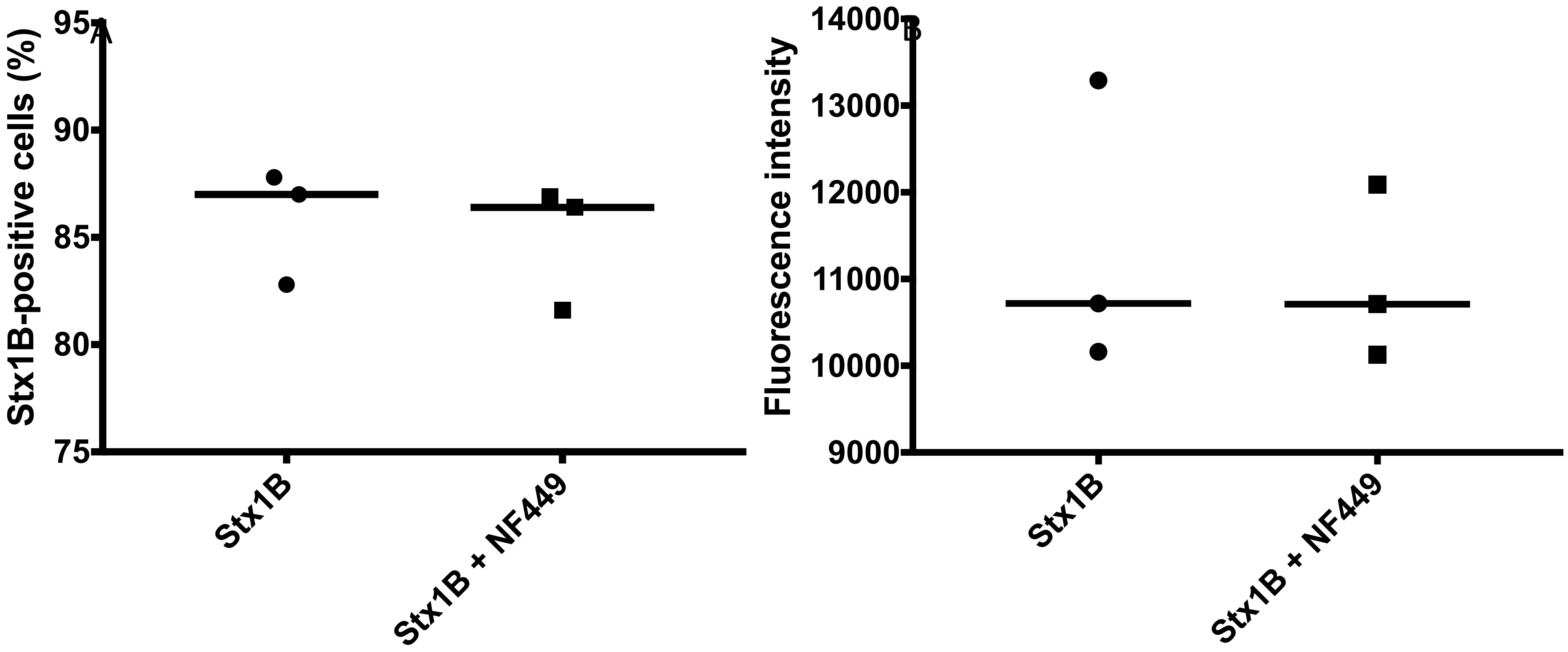

Figure S8: The effect of NF449 on Shiga toxin 1B-binding to HeLa cells

HeLa cells were pretreated with or without NF449 and incubated with Shiga toxin 1B (Stx1B). (A) Cells were analyzed by flow cytometry for the detection of Stx1B-positive cells, showing no statistical difference between NF449-treated and untreated cells. (B) The mean fluorescent intensity was measured, showing no statistical difference regarding Stx1B-positivity between untreated and NF449 treated cells. The bar denotes median percentage of Stx1B-positive cells or fluorescence intensity.

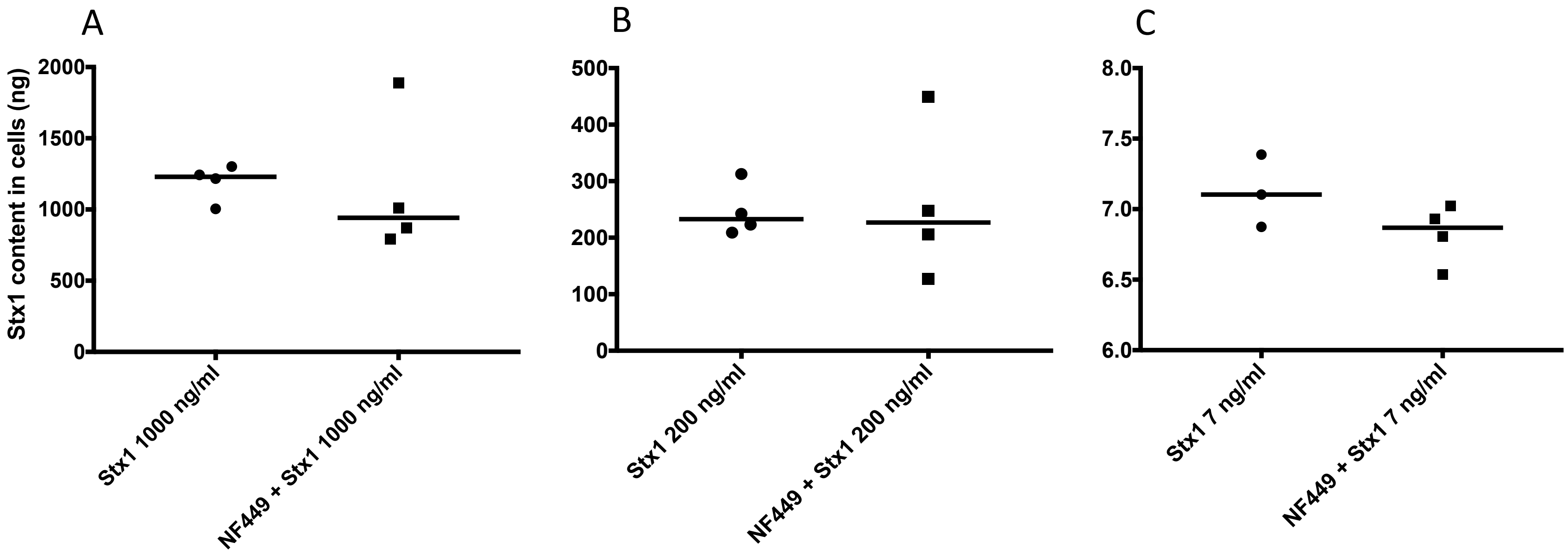

Figure S9: The effect of NF449 on Shiga toxin 1-uptake in HeLa cells
HeLa cells pre-treated with NF449 or PBS vehicle were incubated with Shiga toxin 1 (Stx1) at varying concentrations: (A) 1000, (B) 200 or (C) 7 ng/mL. Results showed that NF449 did not significantly alter the cellular uptake of Stx1. The median is depicted by the bar.
